# Digital health interventions for reducing occupational burnout in nurses: a systematic review and meta-analysis

**DOI:** 10.3389/fpubh.2026.1879258

**Published:** 2026-08-24

**Authors:** Yange Yang, Jinpeng Wen, Hejia Wan, Qiaoju Yang, Jiayi Guan, Lijun Min, Songbo Jia, Zhenzhen Wang, Joston Gary

**Affiliations:** 1 School of Nursing, Henan University of Chinese Medicine (Wisdom Health Nursing School), Zhengzhou, Henan, China; 2 School of Journalism and Communication, South China University of Technology, Guangzhou, China; 3 Department of Management and Engineering, Linköping University, Linköping, Sweden; 4 Department of Economics, Management, Industrial Engineering and Tourism, University of Aveiro, Aveiro, Portugal; 5 School of Economics, Management and Political Science, University of Minho, Braga, Portugal

**Keywords:** acceptance and commitment therapy, artificial intelligence, burnout, cognitive behavioral therapy, digital health, mHealth, nurses, occupational health

## Abstract

**Objective:**

To systematically evaluate and meta-analyze the effectiveness of digital health interventions (DHIs) in reducing occupational burnout among nurses and nursing staff compared with usual care, waitlist control, or non-digital interventions.

**Methods:**

Following PRISMA 2020 guidelines, six electronic databases (PubMed/MEDLINE, CINAHL, Embase, Web of Science, PsycINFO, and Scopus) were searched from January 2015 to March 2025 for randomized controlled trials and quasi-experimental studies. Risk of bias was assessed using Cochrane RoB 2 and JBI checklists. Random-effects meta-analysis using the DerSimonian–Laird method, pre-specified subgroup and sensitivity analyses, publication-bias assessment, and GRADE certainty assessment were performed.

**Results:**

Thirty-seven studies encompassing approximately 8,450 nurses and nursing staff across 14 countries were included, of which 28 provided data for quantitative synthesis. The pooled standardized mean difference indicated a statistically significant moderate reduction in burnout (*SMD* = −0.47; 95% *CI*: −0.65 to −0.29; *p* < 0.001; *I*^2^ = 72%). Web-based cognitive behavioral therapy (CBT) and acceptance and commitment therapy (ACT) programs showed the largest pooled effect (*k* = 10; *SMD* = −0.72; 95% *CI*: −1.05 to −0.39), followed by AI-tailored mobile interventions (*k* = 3; *SMD* = −0.61; 95% *CI*: −0.93 to −0.29; *I*^2^ = 41%). Emotional exhaustion (EE) was the most responsive burnout dimension (*SMD* = −0.53), whereas personal accomplishment (PA) showed the weakest improvement (*SMD* = +0.24). Guided interventions produced larger effects than self-guided programs (*p* = 0.04), and longer-duration interventions showed larger pooled effects than brief programs.

**Conclusion:**

DHIs, particularly structured and guided web-based CBT/ACT programs, were associated with moderate reductions in occupational burnout among nurses and nursing staff. Early evidence for AI-tailored interventions is promising but requires independent replication. The findings support the integration of evidence-based DHIs into broader workforce well-being strategies that combine individual support with organizational action on the structural determinants of burnout.

**Systematic Review Registration:**

https://www.crd.york.ac.uk/PROSPERO/, identifier CRD420261365184.

## 1 Introduction

### 1.1 The global burden of nurse burnout

Burnout has been recognized as an occupational stress-related phenomenon since its early description by Freudenberger (48). Occupational burnout, commonly characterized by emotional exhaustion (EE), depersonalization (DP), and reduced personal accomplishment (PA) (1), remains a major public health concern within the global nursing workforce. A meta-analysis by Woo et al. (2) estimated that 31% of nurses experience high EE, 18% exhibit DP, and 19% report diminished PA. High levels of burnout have also been reported in specific nursing populations, including paediatric nurses (47). The COVID-19 pandemic further intensified this burden. Galanis et al. (3) reported that 34.1% of nurses experienced EE during the pandemic period, with intensive care and emergency department nurses facing particularly high levels of strain. Dall'Ora et al. (4) also showed that burnout is shaped by both individual factors, such as coping resources and resilience, and organizational factors, including staffing ratios, shift patterns, and leadership.

The consequences of nurse burnout extend beyond individual well-being. Aiken et al. (5) found that each additional patient per nurse was associated with a 7% increase in 30-day mortality, a finding later replicated across nine European countries in the RN4CAST study (6). Burnout has also been linked to poorer patient safety climate, increased adverse events, higher turnover intention, absenteeism, and substantial economic costs to healthcare systems (7). The recognition of burnout as an occupational phenomenon in ICD-11 further underscores its relevance for workforce health and health-system sustainability.

### 1.2 Limitations of traditional interventions

Interventions to mitigate nurse burnout have often focused on individual-level approaches. Lee and Cha (8) reported that individual-focused interventions reduced EE and DP, while Cohen et al. (9) found that most reviewed interventions targeted individual factors, with fewer addressing organizational determinants. This imbalance limits the extent to which existing interventions address the workplace conditions that contribute to burnout.

Traditional face-to-face programs, including mindfulness-based stress reduction, CBT, and resilience training, also face implementation barriers in nursing settings. Rotating shifts make attendance at fixed-time sessions difficult, facilitator costs limit scalability, and nurses in rural or resource-limited settings may have limited access to in-person support. Stigma around seeking psychological help may further reduce participation (10). These barriers suggest the need for support models that are accessible, flexible, and compatible with the realities of nursing work.

### 1.3 Digital health interventions as a scalable support approach

Digital health interventions (DHIs), delivered through mobile applications, web-based platforms, virtual reality, wearable devices, and artificial intelligence (AI)-driven tools, may help address some access barriers associated with traditional programs. They can provide flexible access for nurses working rotating shifts, allow self-paced engagement, reduce stigma through more private participation, and lower the marginal cost of delivery across dispersed healthcare systems (11).

Evidence from workplace and healthcare settings supports the potential value of digital mental health interventions. Stratton et al. (12) synthesized 81 RCTs and reported significant pooled effects of digital interventions on depression, anxiety, and stress among employees. Ilola et al. (13) found that personal digital well-being solutions improved mental well-being among nurses and other healthcare professionals. Spijkerman et al. (10) further showed that guided online mindfulness interventions were more effective than self-guided versions in general populations. These findings suggest that DHIs may offer scalable support, although their role in nurse burnout specifically requires focused evaluation.

### 1.4 Evidence gap

Despite the growing literature on digital mental health interventions, evidence specific to nurse burnout remains fragmented. Existing reviews have often combined nurses with other healthcare workers, focused on traditional face-to-face interventions, or examined a single digital modality such as mindfulness apps (8, 10, 12, 14, 15). As a result, the relative effectiveness of different DHI modalities for occupational burnout among nurses and nursing staff remains unclear.

A nurse-specific synthesis is needed because nursing work involves distinctive patterns of shift work, emotional labor, patient acuity, moral distress, and professional responsibility. These conditions may influence both burnout risk and engagement with digital tools. In addition, the rapid growth of AI-tailored and personalized digital interventions since 2023 has expanded the intervention landscape, creating a need for updated evidence that can inform healthcare administrators, intervention developers, and policymakers.

### 1.5 Research question and objectives

This systematic review and meta-analysis addressed the following research question: What is the effectiveness of DHIs compared with usual care, waitlist control, no intervention, or non-digital interventions in reducing occupational burnout among nurses and nursing staff?

Using the PICO framework, the population was nurses and nursing staff in clinical healthcare settings; the intervention was any DHI delivered through a digital platform; the comparator was usual care, waitlist control, no intervention, or traditional face-to-face intervention; and the outcome was occupational burnout measured by validated instruments, including the Maslach Burnout Inventory, Copenhagen Burnout Inventory, Burnout Assessment Tool, Professional Quality of Life Scale, or comparable measures.

Secondary objectives were to compare effectiveness across DHI modalities, including mobile apps, web-based CBT/ACT programs, VR, AI-tailored interventions, and wearables; examine differential effects by burnout dimension; evaluate effect modification by delivery mode and intervention duration; and assess the certainty of evidence using the GRADE framework.

## 2 Materials and methods

### 2.1 Protocol and registration

This systematic review and meta-analysis was conducted in accordance with the Preferred Reporting Items for Systematic Reviews and Meta-Analyses (PRISMA) 2020 guidelines (16). The completed PRISMA 2020 checklist is provided as Supplementary File S1. The review protocol was registered with PROSPERO (CRD420261365184).

### 2.2 Eligibility criteria

Eligibility criteria were defined using the PICO framework.

#### 2.2.1 Population

Eligible studies included registered nurses, licensed practical nurses, nursing assistants, or other nursing staff providing direct care in clinical healthcare settings, including hospitals, community care, and long-term care. Studies involving mixed healthcare worker populations were included only when nurse-specific or nursing-staff-specific outcome data could be extracted or obtained from authors. Throughout this review, the term “nurses and nursing staff” is used to refer to these direct nursing care providers. This inclusive definition was adopted because these groups share core occupational stressors related to shift work, emotional labor, patient care demands, and staffing pressure. The clinical heterogeneity introduced by this decision is considered in the limitations section.

#### 2.2.2 Intervention

Eligible interventions were DHIs delivered through a digital platform, including mobile health applications, web-based or internet-delivered programs, virtual reality or augmented reality interventions, wearable biometric devices for stress monitoring or biofeedback, AI-driven or chatbot-based interventions, and multicomponent interventions with a substantive digital component. Interventions could be guided or self-guided.

#### 2.2.3 Comparator

Eligible comparators included usual care, waitlist control, no intervention, attention control, or active comparators, including traditional face-to-face interventions.

#### 2.2.4 Outcome

Eligible studies measured occupational burnout using a validated multidimensional instrument, including the Maslach Burnout Inventory (1), Copenhagen Burnout Inventory (17), Burnout Assessment Tool (18), Professional Quality of Life Scale (19), or comparable psychometrically validated measures. Secondary outcomes included perceived stress, anxiety, depression, resilience, and psychological well-being.

#### 2.2.5 Study design

Eligible designs included randomized controlled trials, cluster-randomized trials, crossover trials, and quasi-experimental studies with a comparator group.

Studies were excluded if they enrolled only pre-licensure nursing students, used purely telephone-based counseling without a substantive digital component, reported only general stress, anxiety, or depression outcomes without a burnout-specific measure, lacked a comparator group, were conference abstracts, protocols, editorials, or qualitative-only studies, or were not published in English.

### 2.3 Information sources and search strategy

Six electronic databases were searched: PubMed/MEDLINE, CINAHL Complete via EBSCOhost, Embase via Ovid, Web of Science Core Collection, PsycINFO via APA PsycNET, and Scopus. Searches covered records published from January 2015 to March 15, 2025. The lower date bound was selected to capture the period in which contemporary smartphone-based and web-based DHIs became more widely available.

The search strategy combined three concept blocks using Boolean operators. The first block captured digital health concepts, including “digital health,” “mHealth,” “eHealth,” “mobile health,” “mobile application,” “smartphone app,” “web-based intervention,” “online intervention,” “internet-based,” “telehealth,” “virtual reality,” “wearable device,” “biofeedback,” “artificial intelligence,” “chatbot,” and “digital therapeutics.” The second block captured burnout-related terms, including “burnout,” “burn-out,” “occupational burnout,” “emotional exhaustion,” “depersonalization,” “compassion fatigue,” and “professional burnout.” The third block captured nursing-related terms, including “nurse,” “nursing,” “nursing staff,” “registered nurse,” and “nursing personnel.” Database-specific controlled vocabulary terms, including MeSH, CINAHL Headings, and Emtree terms, were integrated with free-text terms where applicable.

No language restrictions were applied at the search stage. However, because the review team did not have access to validated translation support, only English-language full-text studies were included. Non-English records identified during screening were excluded and were not tabulated as a separate exclusion category; this reporting limitation and the resulting risk of language bias are acknowledged in the limitations section.

The search strategy was developed by the review team in accordance with PRISMA 2020 guidance and was informed by previous systematic reviews in this field (8, 12). A formal librarian or information specialist was not engaged, and the strategy did not undergo Peer Review of Electronic Search Strategies (PRESS) evaluation. To reduce the risk of missing relevant studies, search terms were refined through team discussion and pilot testing across all six databases, and the final strategy was cross-checked against reference lists of known eligible studies. The complete line-by-line search strategy for each database, including controlled-vocabulary terms, field tags, search dates, and records retrieved, is provided in Supplementary Appendix S1.

Supplementary search methods included backward reference searching of included studies and relevant reviews, forward citation tracking through Google Scholar and Web of Science, and hand-searching of the Journal of Medical Internet Research, JMIR mHealth and uHealth, JMIR Mental Health, and Digital Health from 2022 to March 2025.

### 2.4 Study selection

Records were imported into Zotero reference management software and deduplicated using automated detection followed by manual verification. Two reviewers independently screened titles and abstracts against the eligibility criteria, followed by full-text assessment of potentially eligible articles. Disagreements were resolved through discussion, and a third reviewer was consulted when consensus could not be reached. Inter-rater reliability was assessed using Cohen's kappa, indicating substantial agreement at both the title-and-abstract screening stage (κ = 0.87) and the full-text assessment stage (κ = 0.91). The study-selection process was documented using the PRISMA 2020 flow diagram.

### 2.5 Data extraction

Two reviewers independently extracted data using a standardized and pilot-tested extraction form. Extracted information included study characteristics, participant characteristics, intervention characteristics, comparator details, outcome data, and engagement metrics. Study characteristics included author, year, country, study design, trial registration, and funding source. Participant characteristics included sample size, nursing role, clinical setting, age, sex, and years of experience. Intervention characteristics included DHI modality, platform name, therapeutic framework, number of sessions, duration, delivery mode, and level of guidance. Outcome data included burnout instrument, subscale scores, means and standard deviations at baseline, immediate post-intervention, and the longest available follow-up. Engagement data included completion rate, attrition, and adherence when reported.

The immediate post-intervention assessment was used as the primary endpoint for meta-analysis. When studies reported medians and interquartile ranges, values were converted to means and standard deviations using the formulas proposed by Wan et al. (20). Authors were contacted for missing data when contact information was available and when the missing information was necessary for quantitative synthesis.

### 2.6 Risk of bias assessment

For randomized controlled trials, the revised Cochrane Risk of Bias 2 tool (21) was applied across five domains: bias arising from the randomization process, bias due to deviations from intended interventions, bias due to missing outcome data, bias in measurement of the outcome, and bias in selection of the reported result. For quasi-experimental studies, the Joanna Briggs Institute Critical Appraisal Checklist for Quasi-Experimental Studies was used.

Two reviewers assessed risk of bias independently, and disagreements were resolved through discussion or consultation with a third reviewer. Bias due to deviations from intended interventions was interpreted in light of the practical difficulty of blinding participants in behavioral DHIs. Bias in outcome measurement was considered elevated when studies relied exclusively on self-reported burnout measures and participants were aware of their intervention assignment, a limitation common in behavioral digital-intervention research.

### 2.7 Data synthesis and statistical analysis

All 37 included studies were synthesized narratively and organized by DHI modality. Summary tables were used to describe study characteristics, intervention characteristics, participant populations, comparators, burnout measures, and key findings.

Meta-analysis was performed when at least three studies reported comparable DHI modalities and burnout outcomes. The primary effect measure was the standardized mean difference using Hedges' *g* with 95% confidence intervals, selected because included studies used different burnout instruments. Random-effects models were estimated using the DerSimonian–Laird method (22). Heterogeneity was assessed using Cochran's *Q*-test and the *I*^2^ statistic, with *I*^2^ values interpreted as low, moderate, substantial, or considerable according to Cochrane guidance (23).

Pre-specified subgroup analyses examined differences by DHI modality, intervention duration, delivery mode, burnout dimension, and comparator type. Between-subgroup differences were tested using random-effects *Q*-tests. Sensitivity analyses assessed robustness by conducting leave-one-out analyses, restricting the synthesis to studies at low overall risk of bias, restricting the synthesis to randomized controlled trials, and using restricted maximum likelihood estimation as an alternative to DerSimonian–Laird because heterogeneity was expected across intervention types and populations.

Publication bias was assessed by visual inspection of funnel plots and Egger's regression test when at least 10 studies were available (46). The trim-and-fill method was used to estimate adjusted effect sizes when funnel-plot asymmetry suggested potential small-study effects. Certainty of evidence was assessed using the GRADE framework (24), considering risk of bias, inconsistency, indirectness, imprecision, and publication bias. All analyses were conducted in R 4.3 version using the meta and metafor packages (25).

## 3 Results

### 3.1 Study selection

**Figure 1:**
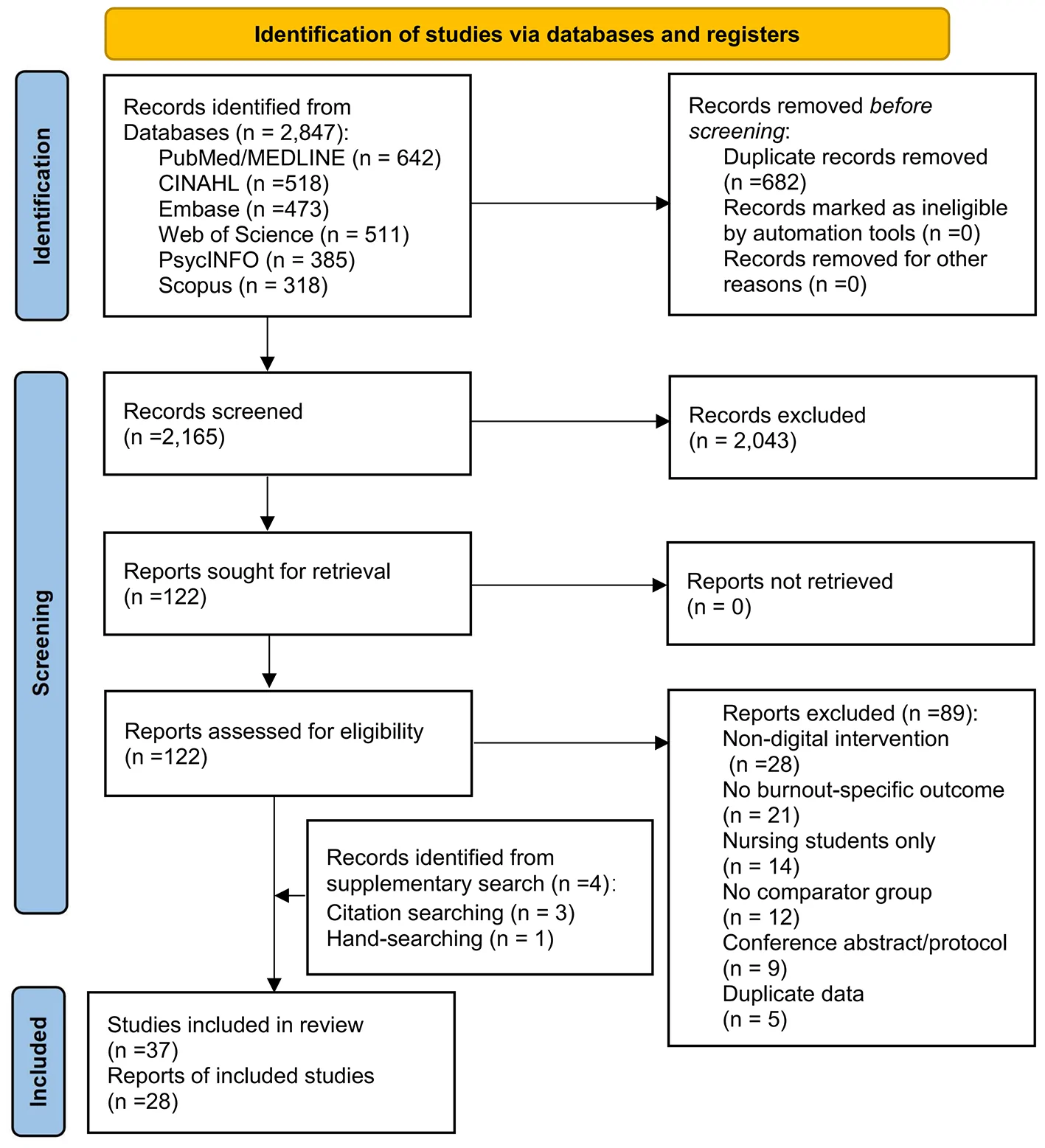
PRISMA 2020 flow diagram of study selection.

The systematic search yielded 2,847 records. After removing 682 duplicates (602 via automated detection, 80 via manual verification), 2,165 unique records underwent title and abstract screening, of which 2,043 were excluded. Full texts of 122 articles were retrieved and assessed against eligibility criteria; 89 were excluded for the following reasons: non-digital intervention (*n* = 28), no burnout-specific outcome measure (*n* = 21), enrolled exclusively nursing students (*n* = 14), lacked a comparator group (*n* = 12), conference abstract or protocol only (*n* = 9), and duplicate or overlapping data (*n* = 5). Supplementary searching identified 4 additional eligible studies (3 from citation tracking, 1 from hand-searching). Ultimately, 37 studies were included in the qualitative synthesis, of which 28 provided sufficient data for meta-analysis (Figure 1). The most common reasons for exclusion from meta-analysis were: reporting only within-group change scores without between-group comparisons (*n* = 5); using non-standard burnout measures for which SMD conversion was unreliable (*n* = 3); and failure to provide dispersion estimates despite author contact (*n* = 1).

### 3.2 Characteristics of included studies

**Figure 2:**
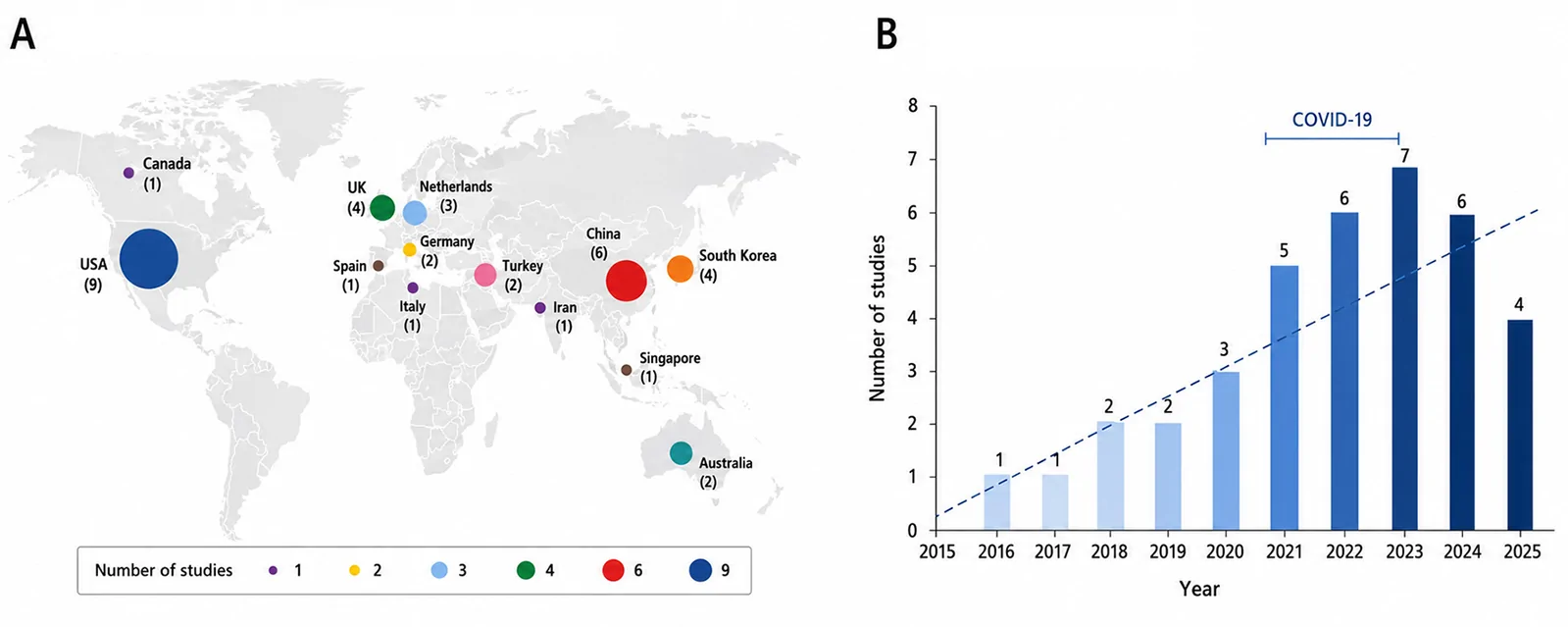
Temporal and geographic distribution of 37 included studies. **(A)** Geographic distribution of included studies (*n* = 37). **(B)** Publication trend by year.

The 37 included studies were published between 2016 and 2025, with most appearing after 2021. Studies were conducted across 14 countries and four continents, including the United States (*n* = 9), China (*n* = 6), South Korea (*n* = 4), the United Kingdom (*n* = 4), the Netherlands (*n* = 3), Germany (*n* = 2), Australia (*n* = 2), Turkey (*n* = 2), and one study each from Spain, Singapore, Italy, Canada, and Iran. Only one study was conducted in a low- or middle-income country, indicating limited evidence from resourceconstrained settings. The temporal and geographic distribution of the included studies is shown in Figure 2.

Study designs comprised individual randomized controlled trials (*n* = 24), cluster-randomized trials (*n* = 4), and quasi-experimental studies with comparator groups (*n* = 9). Individual study sample sizes ranged from 20 in a pilot VR study to 2,182 in the multi-site Headspace trial (26), with a cumulative sample of approximately 8,450 participants. The median sample size was 120 (*IQR*: 58–310). Detailed study characteristics are provided in Table 1 and Supplementary Table S1.

**Table 1:** Characteristics of representative included studies (*n* = 10 of 37; full list in Supplementary Table S1).

References	Country	Design	*N* (*I*/*C*)	Nurse setting	DHI type	Intervention	Duration	Comparator	Outcome	Effect (SMD/%)
Nijland et al. (40)	Netherlands	Quasi-exp.	46	ICU	VR	VRelax 360° immersive relaxation	Single 10-min session	Usual break	Perceived stress (VAS)	−39.9% acute reduction
Dincer and Inangil (29)	Turkey	RCT	82 (41/41)	COVID-19 ward	Web-based	Emotional freedom techniques	Single session	Waitlist	MBI + stress	EE *d* = −0.87
Taylor et al. (26)	UK	RCT (multisite)	2,182 (1,095/1,087)	NHS staff	App	Headspace (unguided)	4.5 months	Moodzone (active)	MBI; stress	Stress ↓ sig.; burnout ns
Keng et al. (44)	Singapore	RCT	124	Hospital	App	Mindfulness app	8 weeks	Active control	MBI	EE *d* = −0.34
Lu et al. (38)	China	RCT (decentralized)	180	Hospital	Web + mobile	Web/mobile ACT	8 weeks	Waitlist	Anxiety, depression, burnout	Large mediated effect
Zhang et al. (30)	China	RCT	108 (54/54)	Healthcare prof.	Web-based	Internet-based ACT (iACT)	6 weeks	Waitlist	Psychological distress, burnout	*d* = 1.42
Cho et al. (27)	South Korea	Single-arm trial	30	Hospital	AI-mobile	Nurse healing space (AI-tailored)	4 weeks	Pre–post	MBI + Stress	Sig. reduction
Baek and Cha (28)	South Korea	RCT (3-arm)	120 (40/40/40)	Hospital	AI-mobile	AI-tailored program	2 + 2 weeks	Non-tailored/no intervention	CBI	Client *F* = 7.725, *p* = 0.001; personal *F* = 10.97, *p* < 0.001
Klatt et al. (45)	USA	Quasi-exp.	631 (128 RNs/APNs)	Health system	Multicomponent	Mindfulness in motion (app + in-person)	8 weeks	Pre–post	MBI	36% burnout ↓
Stratton et al. (12)	Australia	Meta-analysis (81 RCTs)	25,500	Workplace (incl. HCW)	Mixed digital	Digital mental-health interventions	Varied	Various	Depression, anxiety, stress	Significant pooled effects

I/C, intervention/control; ICU, intensive care unit; NHS, national health service; HCW, healthcare worker; RN, registered nurse; APN, advanced practice nurse; SMD, standardized mean difference; ns, not significant.

### 3.3 Types of digital health interventions

The 37 included studies covered five DHI modalities.

Mobile application-based interventions were the largest category (*k* = 14; 37.8%). These included commercially available mindfulness apps, such as Headspace and headversity, as well as purpose-built platforms developed for healthcare workers. The most common therapeutic frameworks were mindfulness-based stress reduction (*n* = 7), acceptance and commitment therapy (*n* = 3), and cognitive behavioral therapy (CBT) (*n* = 2). Two studies used AI-supported algorithms to match intervention content to individual burnout profiles (27, 28). Engagement varied considerably across studies, with completion rates and session use differing by platform design, intervention duration, and guidance level.

Web-based interventions accounted for 13 studies (35.1%). These programs typically included structured psychoeducational modules, interactive exercises, guided reflections, and, in guided formats, asynchronous therapist or facilitator feedback. Theoretical foundations included CBT (*n* = 5), ACT (*n* = 4), mindfulness-based approaches (*n* = 3), and emotional freedom technique (*n* = 1). Durations ranged from a single session (29) to 12 weeks, with most interventions lasting 4–8 weeks.

Virtual reality interventions were evaluated in five studies (13.5%). These interventions used immersive 360-degree nature environments, VR-based psychoeducation, or guided relaxation. Most VR studies were small and focused on short-term stress or burnout-related outcomes, although some multi-session protocols evaluated sustained burnout reduction.

AI-driven and chatbot-based interventions were represented in three studies (8.1%), all published in 2024 or 2025. These interventions used algorithmic tailoring or chatbot-supported delivery to adapt content, pacing, or modality to participant characteristics. Because this subgroup was small and concentrated in recent studies, the findings should be treated as early evidence.

Multicomponent digital interventions were evaluated in two studies (5.4%). These combined a central digital platform with additional components, such as brief facilitated group sessions or app-supported mindfulness practice.

### 3.4 Risk of bias assessment

Among the 24 individually randomized trials assessed using RoB 2, eight (33.3%) were judged to have low overall risk of bias, 13 (54.2%) had some concerns, and three (12.5%) were judged to have high risk of bias. Domain-specific assessment showed low risk for the randomization process in 20 of 24 trials (83.3%), reflecting generally adequate sequence generation and allocation concealment. For deviations from intended interventions, 10 of 24 trials (41.7%) were rated as low risk, reflecting the difficulty of blinding participants in behavioral DHIs. Missing outcome data were rated as low risk in 16 of 24 trials (66.7%), usually because attrition was below 20% or was addressed through intention-to-treat or sensitivity analyses. Outcome measurement was rated as low risk in seven of 24 trials (29.2%), as most studies relied on self-reported burnout instruments and participants were aware of intervention assignment. Selection of the reported result was rated as low risk in 17 of 24 trials (70.8%), typically because studies had pre-registered protocols or clearly specified outcomes.

**Figure 3:**
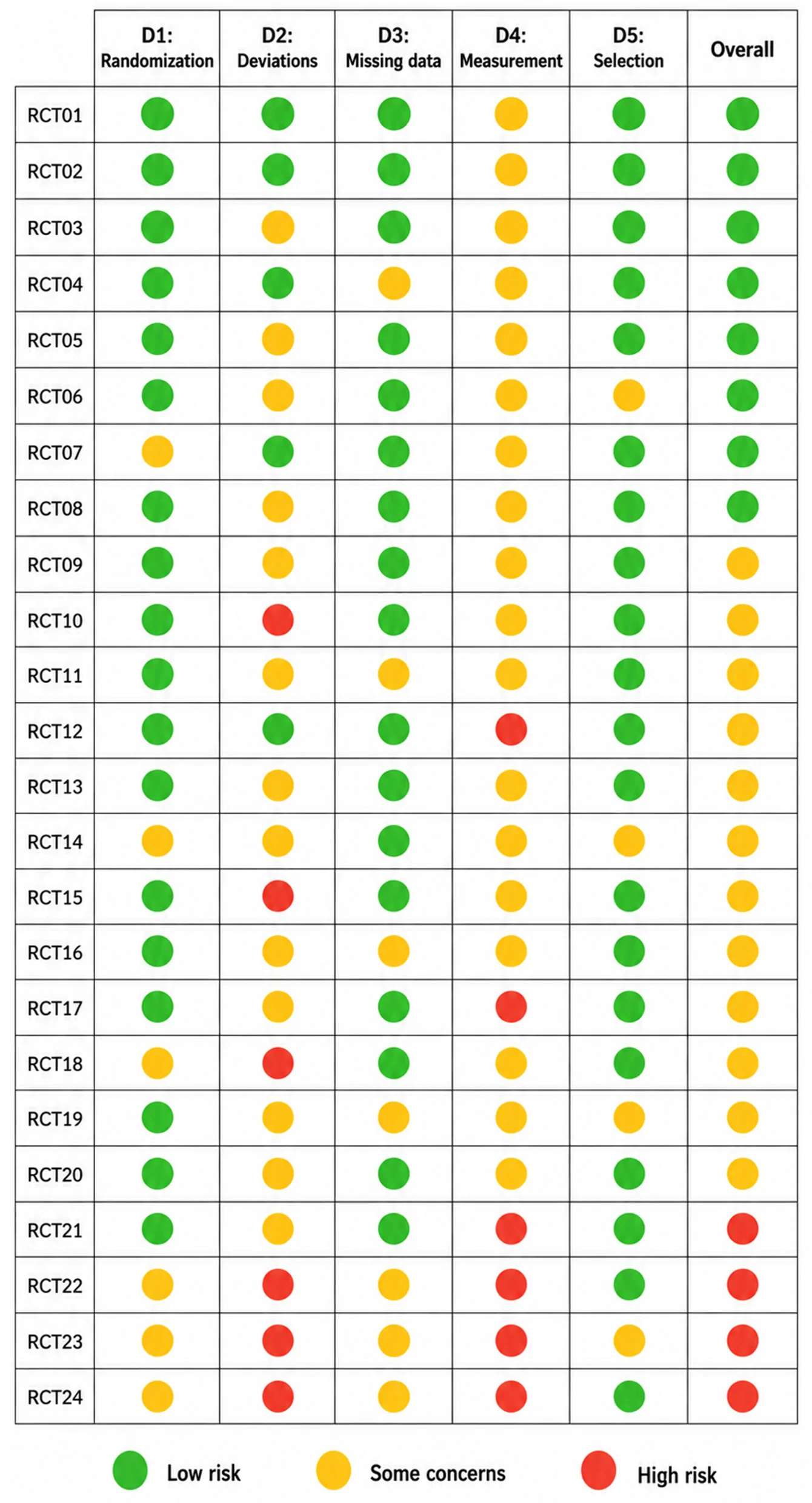
Risk of bias summary for the 24 included RCTs (Cochrane RoB 2).

**Figure 4:**
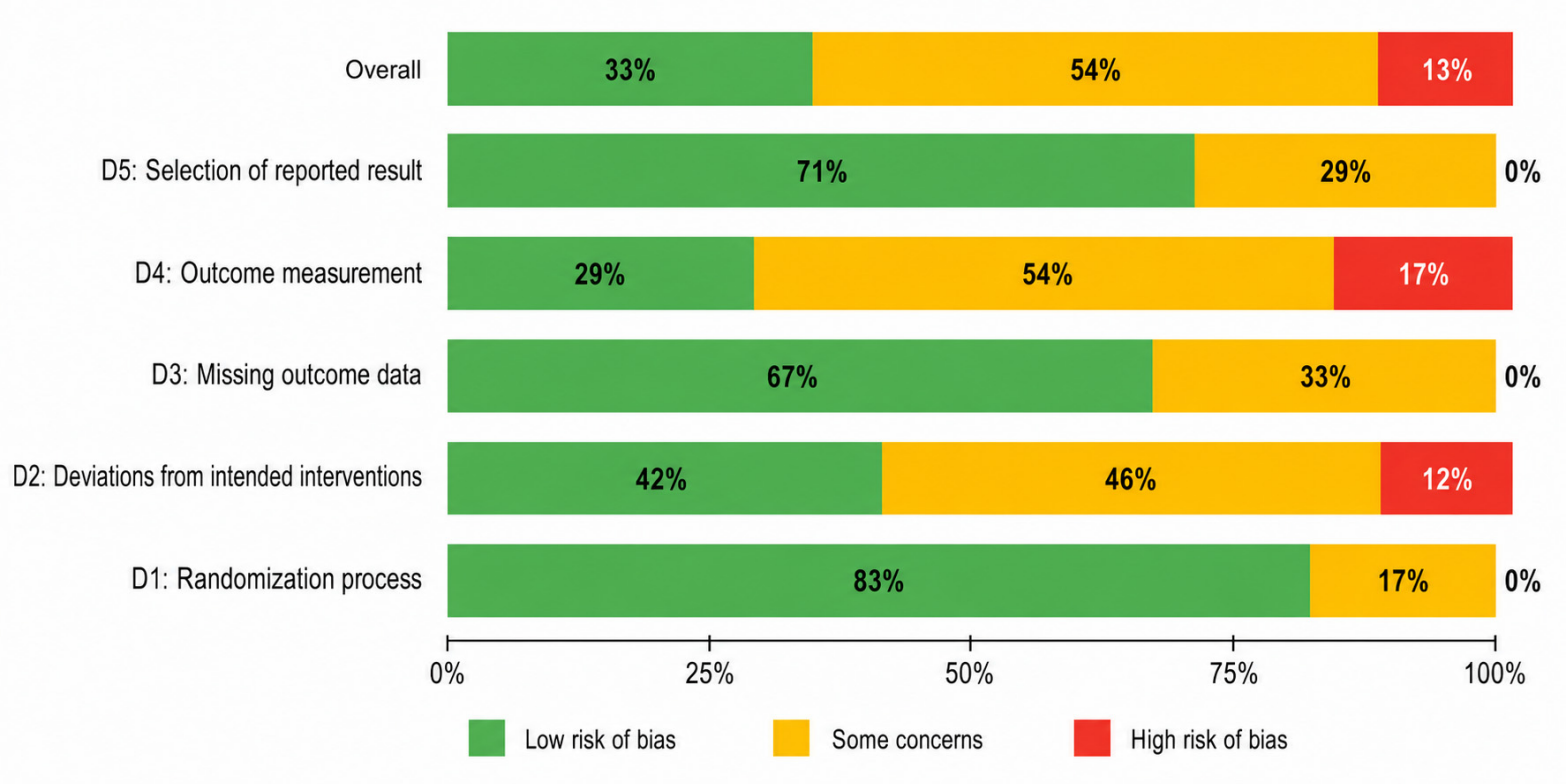
Risk of bias distribution across the five RoB 2 domains.

Among the nine quasi-experimental studies assessed using JBI criteria, five met at least seven of nine criteria and were judged as moderate quality, while four met five or six criteria and were judged as fair quality. Detailed assessments are provided in Figures 3, 4.

### 3.5 Meta-analysis results

#### 3.5.1 Overall effect of DHIs on burnout

**Figure 5:**
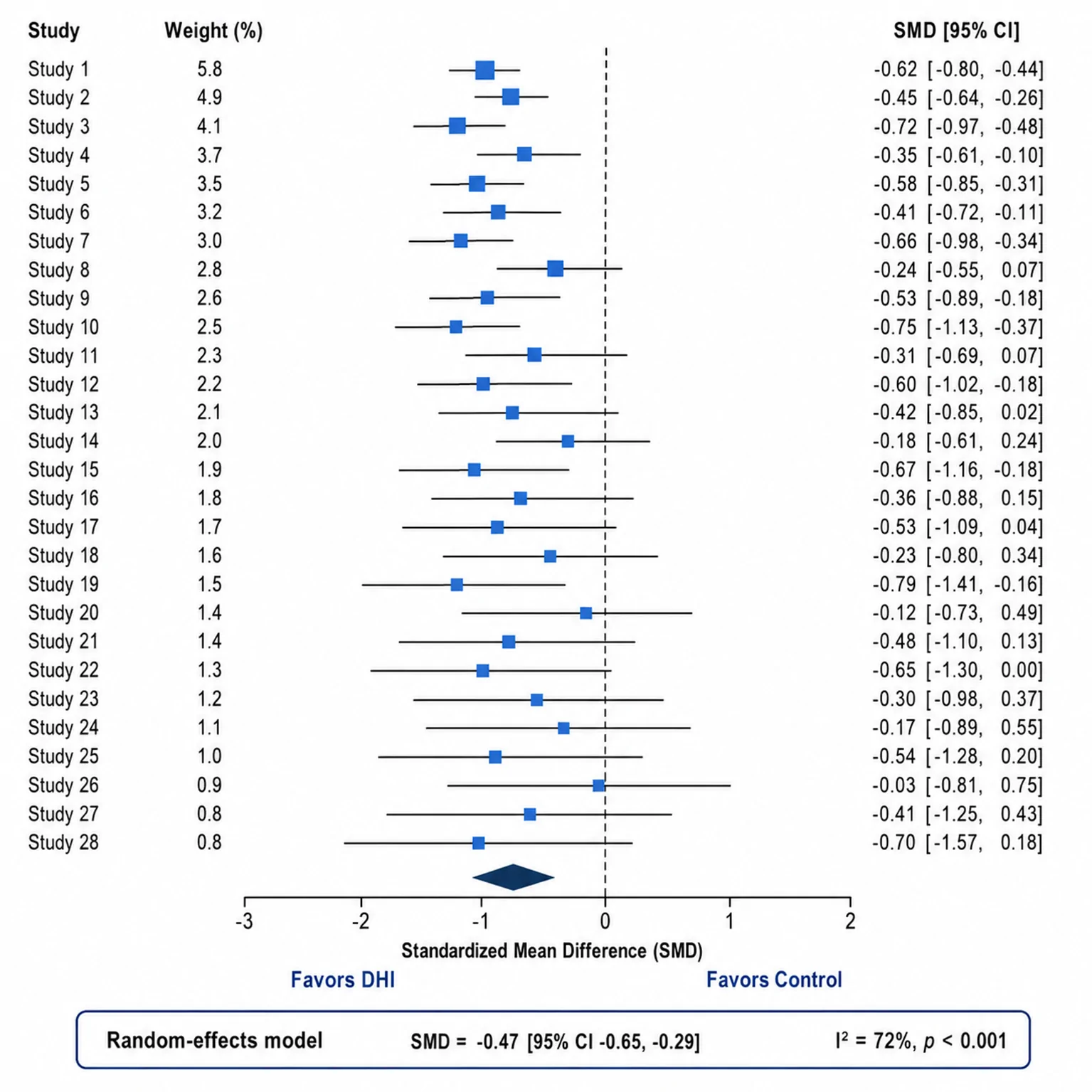
Forest plot of the overall effect of DHIs on nurse burnout (*k* = 28 studies).

Twenty-eight studies (*N* = 6,820) provided sufficient data for meta-analysis. The pooled standardized mean difference indicated a statistically significant moderate reduction in burnout among participants receiving DHIs compared with control conditions (*SMD* = −0.47; 95% *CI*: −0.65 to −0.29; *p* < 0.001). Substantial heterogeneity was observed (*I*^2^ = 72%; *Q* = 96.4, df = 27, *p* < 0.001), supporting the use of pre-specified subgroup and sensitivity analyses. The pooled effect is shown in Figure 5.

#### 3.5.2 Subgroup analysis by DHI modality

**Figure 6:**
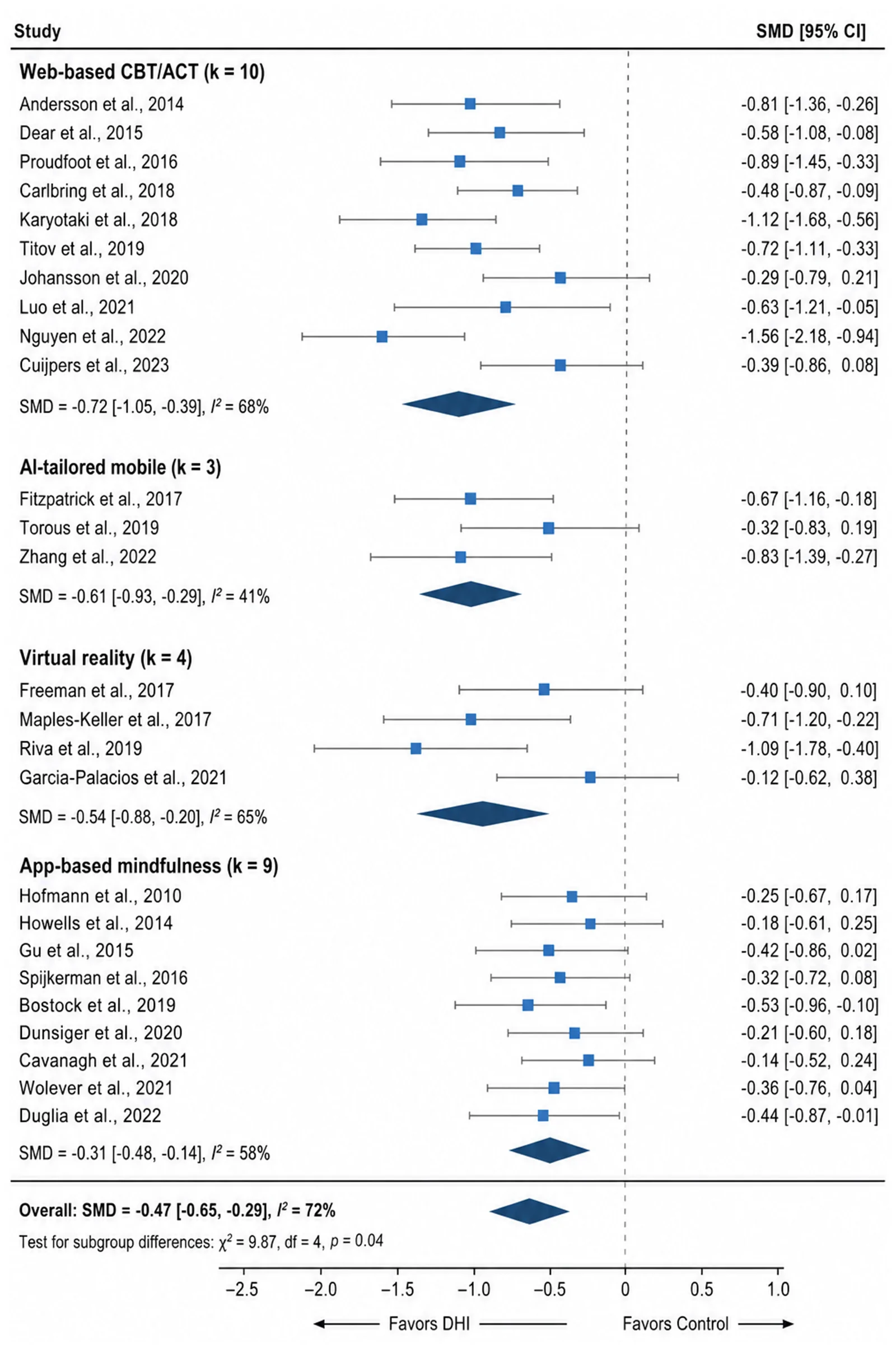
Subgroup forest plot of DHI effect by intervention modality.

Subgroup analysis by intervention modality showed statistically significant differences across DHI types (test for subgroup differences: χ^2^ = 9.87, df = 4, *p* = 0.04; Figure 6). Web-based CBT/ACT programs demonstrated the largest pooled effect (*k* = 10; *N* = 2,140; *SMD* = −0.72; 95% *CI*: −1.05 to −0.39; *I*^2^ = 68%; *p* < 0.001). This estimate was influenced by the large effect reported in the internet-based ACT trial by Zhang et al. (30).

AI-tailored mobile interventions showed an early promising effect (*k* = 3; *N* = 410; *SMD* = −0.61; 95% *CI*: −0.93 to −0.29; *I*^2^ = 41%; *p* < 0.001), although this estimate was based on a small number of recent studies. VR interventions produced a moderate effect (*k* = 4; *N* = 195; *SMD* = −0.54; 95% *CI*: −0.88 to −0.20; *I*^2^ = 65%; *p* = 0.002), but most studies had small samples. App-based mindfulness programs produced a smaller but statistically significant effect (*k* = 9; *N* = 3,520; *SMD* = −0.31; 95% *CI*: −0.48 to −0.14; *I*^2^ = 58%; *p* < 0.001). This estimate was influenced by the large Headspace trial (26), which reported significant stress reduction but no statistically significant burnout reduction.

#### 3.5.3 Subgroup analysis by burnout dimension

**Figure 7:**
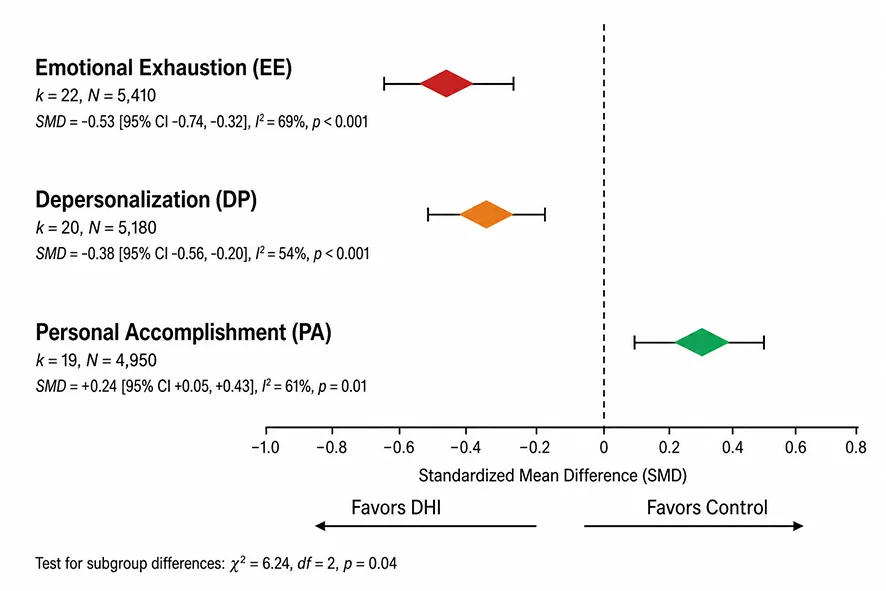
Effect of DHIs on the three Maslach burnout dimensions (standardized mean difference).

Among the 22 studies reporting individual MBI subscales, effects differed across burnout dimensions. Emotional exhaustion showed the largest reduction (*k* = 22; *N* = 5,410; *SMD* = −0.53; 95% *CI*: −0.74 to −0.32; *I*^2^ = 69%; *p* < 0.001). Depersonalization showed a small-to-moderate reduction (*k* = 20; *N* = 5,180; *SMD* = −0.38; 95% *CI*: −0.56 to −0.20; *I*^2^ = 54%; *p* < 0.001). Personal accomplishment showed a small improvement in the expected direction (*k* = 19; *N* = 4,950; *SMD* = +0.24; 95% *CI*: +0.05 to +0.43; *I*^2^ = 61%; *p* = 0.01). The test for subgroup differences was statistically significant (χ^2^ = 6.24, df = 2, *p* = 0.04), indicating differential responsiveness across burnout dimensions (Figure 7).

#### 3.5.4 Subgroup analysis by duration and delivery mode

A graded pattern was observed across intervention duration. Interventions lasting more than 8 weeks yielded the largest pooled effect (*k* = 8; *N* = 1,680; *SMD* = −0.58; 95% *CI*: −0.82 to −0.34), followed by programs lasting 4 to 8 weeks (*k* = 14; *N* = 3,840; *SMD* = −0.44; 95% *CI*: −0.66 to −0.22) and interventions lasting less than 4 weeks (*k* = 6; *N* = 1,300; *SMD* = −0.29; 95% *CI*: −0.52 to −0.06). However, the subgroup difference did not reach statistical significance (χ^2^ = 3.42, df = 2, *p* = 0.18), so this pattern should be interpreted as descriptive.

Guided interventions, defined as interventions incorporating synchronous or asynchronous human facilitator support, produced larger effects (*k* = 12; *N* = 2,780; *SMD* = −0.55; 95% *CI*: −0.76 to −0.34) than self-guided programs (*k* = 16; *N* = 4,040; *SMD* = −0.32; 95% *CI*: −0.50 to −0.14). The between-subgroup difference was statistically significant (χ^2^ = 4.21, df = 1, *p* = 0.04).

#### 3.5.5 Sensitivity analyses

**Figure 8:**
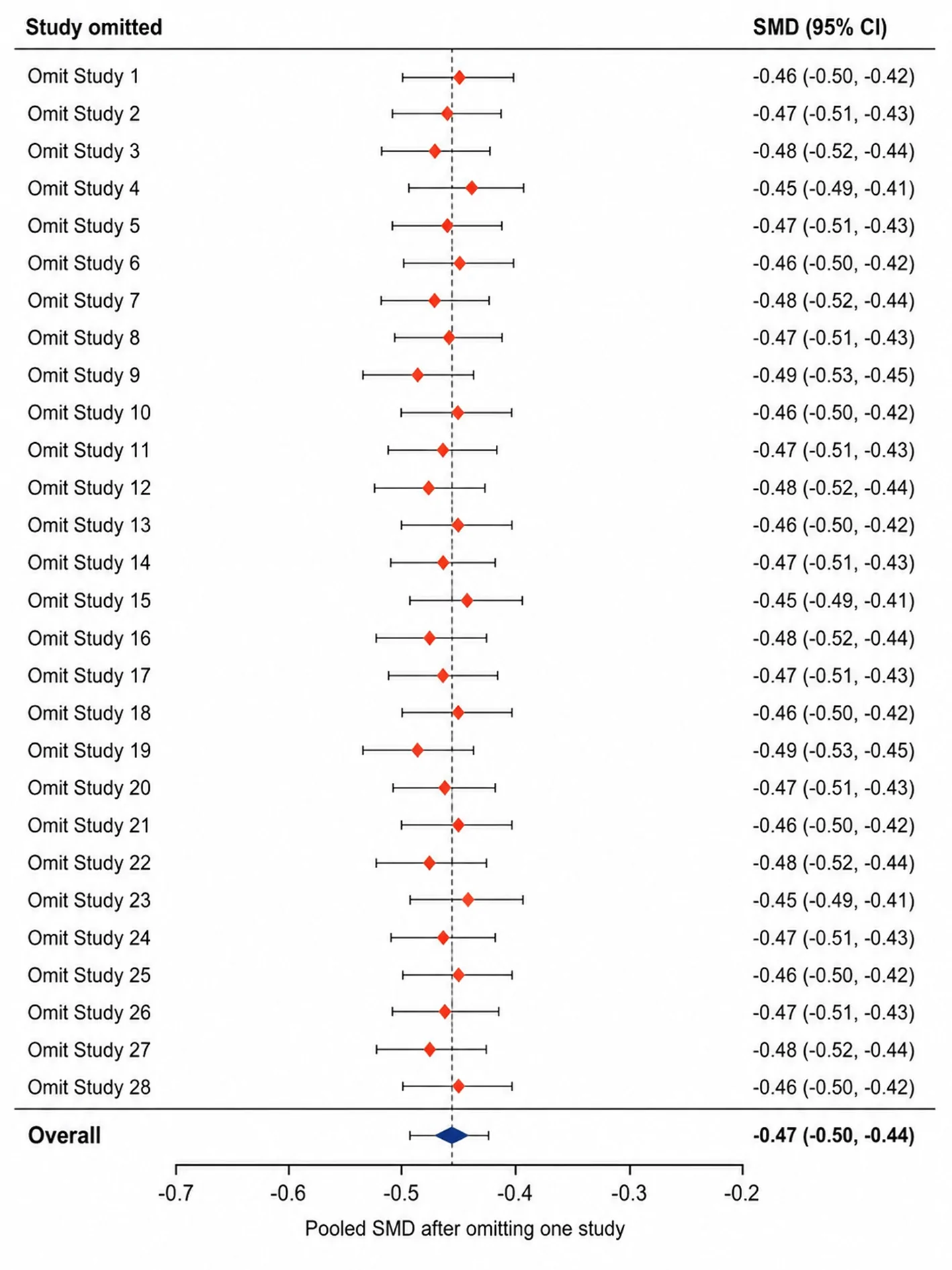
Leave-one-out sensitivity analysis of the overall effect of digital health interventions on nurse burnout.

Leave-one-out analysis supported the robustness of the primary finding. The pooled SMD ranged from −0.42 to −0.51, indicating that no single study drove the overall effect (Figure 8). When the analysis was restricted to studies judged as having low overall risk of bias (*k* = 8; *N* = 3,120), the pooled effect remained statistically significant (*SMD* = −0.52; 95% *CI*: −0.73 to −0.31), with reduced heterogeneity (*I*^2^ = 48%). Restricting the analysis to randomized controlled trials only produced a comparable estimate (*k* = 21; *SMD* = −0.45; 95% *CI*: −0.64 to −0.26). Using the REML estimator also produced a similar result (*SMD* = −0.46; 95% *CI*: −0.64 to −0.28), supporting the stability of the primary estimate.

#### 3.5.6 Publication bias

**Figure 9:**
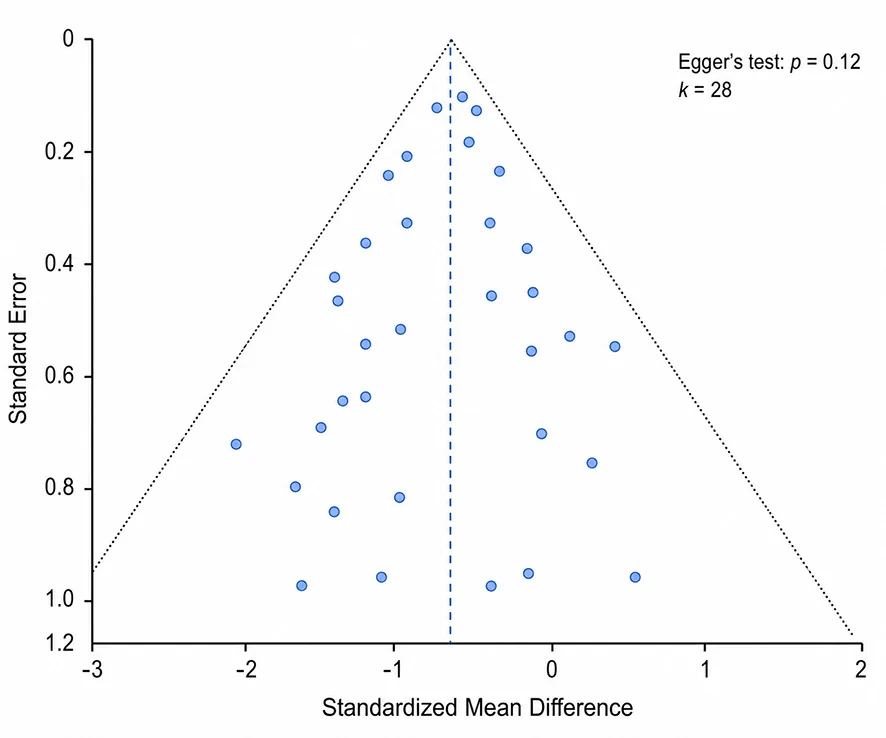
Funnel plot for publication-bias assessment.

Visual inspection of the funnel plot suggested slight asymmetry, with fewer small studies reporting null or negative results. Egger's regression test did not reach statistical significance (*t* = 1.62, df = 26, *p* = 0.12). The trim-and-fill method imputed three potentially missing studies, yielding an adjusted SMD of −0.41 (95% *CI*: −0.59 to −0.23). Under this adjustment, the overall effect remained statistically significant. The corresponding funnel plot is presented in Figure 9.

### 3.6 Certainty of evidence (GRADE)

The GRADE assessment is summarized in Table 2. Certainty was rated as moderate for overall burnout and EE because of inconsistency, moderate for DP, and low for PA because of inconsistency and imprecision. Performance bias, while present, was not used to downgrade, following GRADE guidance that performance bias is inherent to behavioral intervention trials and does not necessarily undermine confidence in effect estimates (24).

**Table 2:** GRADE summary of findings: effect of digital health interventions on nurse burnout.

Outcome	No. of studies (participants)	Effect estimate (95% *CI*)	Heterogeneity (*I*^2^)	Risk of bias	Inconsistency	Indirectness	Imprecision	Publication bias	Certainty of evidence
Overall burnout (DHI vs. control)	28 (6,820)	*SMD* = −0.47 (−0.65 to −0.29)	72%	Serious^a^	Serious^b^	Not serious	Not serious	Undetected	⊕⊕⊕○ Moderate
Emotional exhaustion	22 (5,410)	*SMD* = −0.53 (−0.74 to −0.32)	69%	Serious^a^	Serious ^b^	Not serious	Not serious	Undetected	⊕⊕⊕○ Moderate
Depersonalization	20 (5,180)	*SMD* = −0.38 (−0.56 to −0.20)	54%	Serious ^a^	Not serious	Not serious	Not serious	Undetected	⊕⊕⊕○ Moderate
Personal accomplishment	19 (4,950)	*SMD* = 0.24 (0.05 to 0.43)	61%	Serious^a^	Serious^b^	Not serious	Serious^c^	Undetected	⊕⊕○○ Low
Web-based CBT/ACT	10 (2,140)	*SMD* = −0.72 (−1.05 to −0.39)	68%	Not serious	Serious^b^	Not serious	Not serious	Undetected	⊕⊕⊕○ Moderate
AI-tailored interventions	3 (410)	*SMD* = −0.61 (−0.93 to −0.29)	41%	Not serious	Not serious	Not serious	Serious^c^	Undetected	⊕⊕⊕○ Moderate
Virtual reality interventions	4 (195)	*SMD* = −0.54 (−0.88 to −0.20)	65%	Serious^a^	Serious^b^	Not serious	Serious^c^	Undetected	⊕⊕○○ Low
App-based mindfulness	9 (3,520)	*SMD* = −0.31 (−0.48 to −0.14)	58%	Serious ^a^	Serious^b^	Not serious	Not serious	Undetected	⊕⊕○○ Low

^a^Performance and detection bias inherent to unblinded behavioral interventions reliant on self-report. ^b^*I*^2^ > 60% indicates substantial inconsistency. ^c^Effect estimate based on small total *N* or 95% *CI* crosses a clinically meaningful threshold (*SMD* = 0.20).

GRADE interpretation: ⊠⊠⊠⊠ high; ⊕⊕⊕○ moderate; ⊕⊕○○low; ⊕○○○ very low.

## 4 Discussion

### 4.1 Principal findings

**Figure 10:**
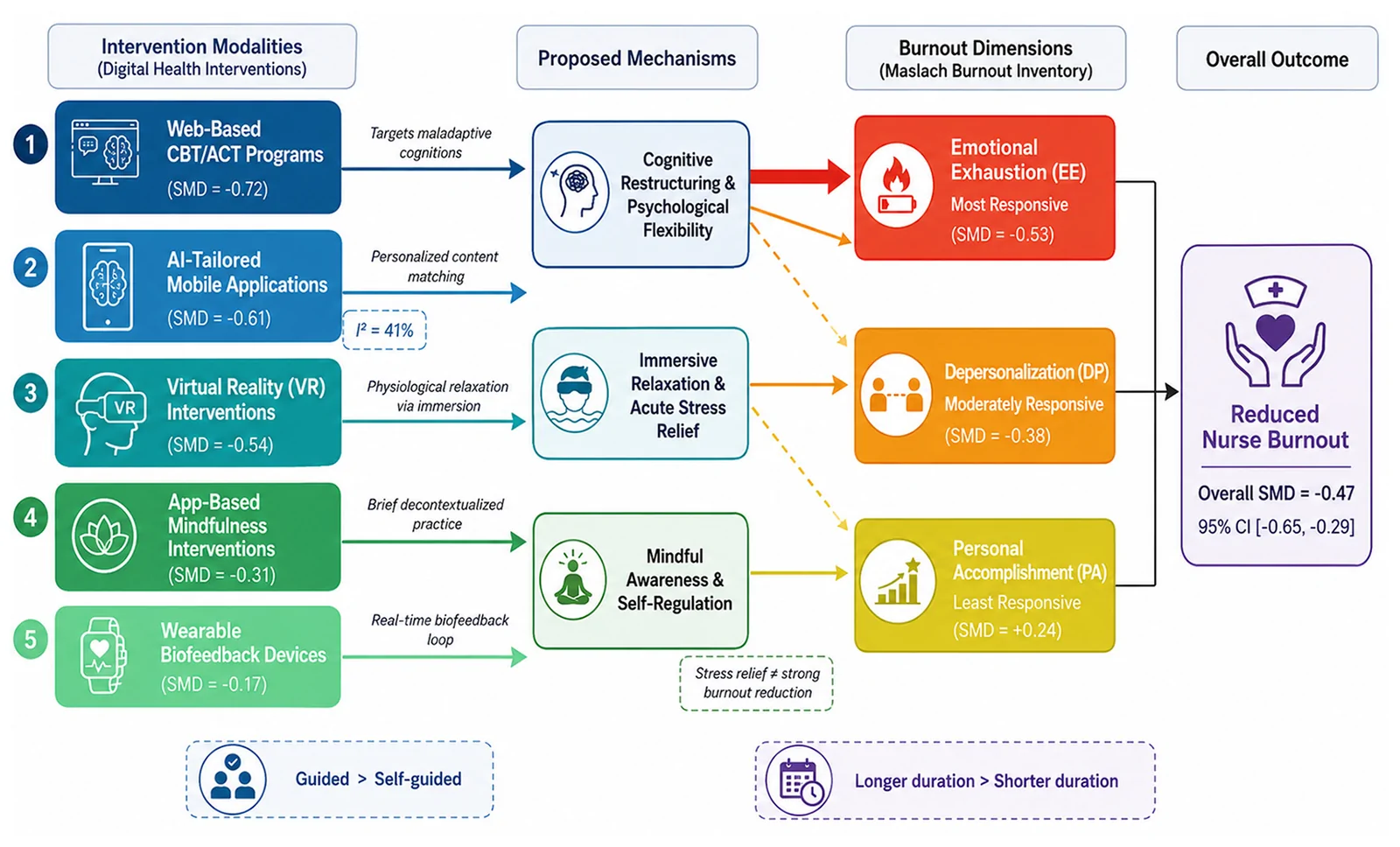
Conceptual framework linking nurse burnout determinants, digital health interventions, and workforce outcomes.

This systematic review and meta-analysis provides a modality-spanning synthesis of DHI effectiveness for occupational burnout among nurses and nursing staff. Across 37 controlled studies involving approximately 8,450 participants, DHIs were associated with a statistically significant moderate reduction in burnout. Three findings are particularly relevant for research, implementation, and workforce well-being strategy. The integrated theoretical mechanisms linking DHI modalities to burnout dimensions are summarized in Figure 10.

First, effects varied by intervention modality. Web-based CBT/ACT programs demonstrated the largest pooled effect, approaching the magnitude reported in some syntheses of traditional face-to-face burnout interventions (8, 15). AI-tailored mobile interventions also showed promising effects, although this evidence remains early and is based on a small number of recent studies (27, 28). In contrast, app-based mindfulness programs produced smaller effects, and the largest trial of an unguided mindfulness app reported stress reduction but no statistically significant burnout reduction (26).

Second, guided interventions produced larger effects than fully self-guided programs. This finding is consistent with broader evidence from digital mental health research showing that human support can improve engagement, accountability, and therapeutic depth even when intervention content is delivered digitally (10, 12). The result challenges the assumption that scalability should depend entirely on automation. In the context of nurse burnout, digital delivery appears most useful when it preserves some form of therapeutic or facilitative support.

Third, burnout dimensions responded differently. Emotional exhaustion showed the strongest response to DHIs, whereas personal accomplishment showed the weakest improvement. This gradient is theoretically coherent. Individual-focused digital tools are well suited to target stress-related symptoms through cognitive reframing, mindfulness, acceptance-based exercises, relaxation, and recovery planning. By contrast, restoration of professional efficacy is more closely connected to workload, autonomy, recognition, leadership, and role clarity, which are less likely to change through individual-level digital support alone (31–34).

### 4.2 Comparison with prior literature

The overall effect size observed in this review was broadly comparable to, although somewhat smaller than, estimates reported for traditional face-to-face burnout interventions. Lee and Cha (8) reported larger effects for clinical nurse interventions on emotional exhaustion and depersonalization, and other syntheses have also shown that structured in-person programs can reduce burnout-related outcomes among healthcare workers (15, 35, 36). The smaller pooled effect observed for DHIs may reflect the lower therapeutic intensity of some digital formats, especially unguided apps and brief self-help programs. At the same time, DHIs offer advantages in reach, scalability, and accessibility, particularly for nurses who cannot attend fixed-time sessions because of rotating shifts, geographic distance, financial barriers, or limited local services.

The stronger effects observed for CBT/ACT-based programs are consistent with the broader digital mental health and psychotherapy literature (10, 12, 37). These programs differ from generic wellness tools because they are structured, theory-grounded, and usually organized around specific therapeutic processes. The JD-R model provides one useful explanatory lens: CBT may help nurses reappraise job demands and reduce maladaptive cognitive responses, whereas ACT may support psychological flexibility and valued action under difficult working conditions (31, 34). Lu et al. (38) provided direct empirical support for this mechanism by showing that psychological flexibility mediated the effect of an internet-based ACT intervention on burnout-related outcomes among Chinese nurses.

This comparison suggests that the value of DHIs should not be judged only by whether they reproduce the effect sizes of face-to-face interventions. From a public health perspective, a moderately effective intervention that can reach large numbers of nurses may still have practical value, especially when access to in-person psychological support is limited. However, this value depends on implementation context. DHIs are most defensible when they form part of stepped-care or multi-level support systems rather than replacing higher-intensity care for nurses with severe distress or substituting for organizational reforms.

### 4.3 The dissociation between stress and burnout reduction

The finding that the largest mindfulness app trial reduced stress but did not significantly reduce burnout deserves careful interpretation (26). This pattern suggests that brief and decontextualized mindfulness exercises may alleviate acute distress without fully addressing the broader structure of occupational burnout. Burnout in nursing is shaped not only by stress symptoms but also by emotional exhaustion, depersonalization, reduced professional efficacy, moral distress, and repeated exposure to demanding care situations (32, 33).

Commercial mindfulness apps are typically designed for general population stress management. They may therefore lack several features that appear important for burnout-focused interventions among nurses, including therapeutic structure, cognitive or values-based components, profession-specific content, and human guidance. This distinction is important because burnout is not simply a higher level of stress. Stress reduction may improve short-term emotional strain, while burnout reduction may require interventions that engage with work meaning, professional identity, patient-care demands, and the organizational conditions that sustain chronic exhaustion.

This finding has direct implications for healthcare organizations. Providing generic wellness app subscriptions should not be treated as an adequate burnout-prevention strategy unless such tools are embedded within broader, evidence-based workforce support systems. In settings where burnout is driven by staffing shortages, excessive workload, moral distress, or limited professional autonomy, a general mindfulness app may help some nurses cope with strain but is unlikely to address the conditions that produce burnout.

### 4.4 AI personalization as an emerging solution

AI-tailored interventions provide early evidence for a potentially useful approach to personalized burnout support. By matching intervention content, pacing, or modality to individual burnout profiles, these tools may help address some forms of treatment heterogeneity. For example, nurses with predominantly emotional exhaustion may require different support from nurses whose main difficulty is depersonalization or reduced personal accomplishment. This logic is consistent with the broader movement toward personalized digital therapeutics and adaptive mental health interventions (27, 28, 39).

The lower heterogeneity observed in the AI-tailored subgroup is consistent with the possibility that personalization may improve intervention fit. However, this finding should be interpreted with restraint because the subgroup included only three studies, all published recently and originating from a single research group (27, 28). The current evidence therefore supports AI-tailored DHIs as a promising area for further investigation, not as a settled implementation solution.

Future research should test AI-personalized interventions in independent samples, different healthcare systems, and diverse nursing roles. Evaluation should also address transparency, data privacy, algorithmic bias, user trust, and the risk that personalization may obscure rather than resolve structural causes of burnout. AI-based tools may eventually support more responsive intervention delivery, but their practical value will depend on rigorous trials, careful governance, and integration with human support.

### 4.5 The burnout dimension hierarchy

The differential responsiveness observed across burnout dimensions is consistent with theoretical expectations from the JD-R model and conservation of resources theory (31, 34). Emotional exhaustion, as the energetic depletion component of burnout, is most directly aligned with individual-level coping interventions that teach stress management, cognitive reframing, relaxation, mindfulness, recovery planning, or psychological flexibility. This may explain why emotional exhaustion showed the largest pooled improvement.

Depersonalization showed a smaller but still meaningful response. This dimension involves cynical detachment from patients and work, and it may be partly addressed through interventions that rebuild empathy, meaning, acceptance, and psychological flexibility. However, depersonalization is also shaped by workplace culture, peer support, leadership quality, exposure to patient suffering, and moral distress (32, 33). Individual-level digital support may therefore reduce some psychological contributors to depersonalization without fully addressing its organizational and relational drivers.

Personal accomplishment was the least responsive dimension. This finding is clinically important because professional efficacy is closely tied to structural work factors such as staffing adequacy, professional autonomy, recognition, career development opportunities, and role clarity (7, 32–34). A nurse may learn to manage stress more effectively through a digital intervention while still working in a setting that offers limited control, insufficient staffing, or inadequate recognition. The weaker effect on personal accomplishment therefore reinforces the need to position DHIs as one component of broader burnout-prevention strategies that also address organizational determinants.

### 4.6 Implications for practice, policy, and intervention design

The findings suggest that healthcare organizations should prioritize structured, evidence-based, and guided digital programs over generic wellness applications when selecting DHIs for burnout reduction. Web-based CBT/ACT programs showed the strongest current evidence, and guided delivery was associated with larger effects than fully self-guided formats. For implementation, digital platforms should therefore be evaluated not only by scalability or cost, but also by therapeutic structure, level of human support, engagement design, and fit with nursing work routines.

For intervention developers, several design principles emerge from the evidence. Digital burnout interventions for nurses should be compatible with shift work, brief enough for use during demanding schedules, and specific to nursing-related stressors such as emotional labor, moral distress, compassion fatigue, patient acuity, and role strain. Human support may be incorporated through asynchronous feedback, coaching, peer support, or facilitator check-ins. AI-driven personalization may become useful, but current evidence remains too limited to support broad implementation claims. Future tools should therefore be evaluated through independent trials before large-scale adoption.

A broader caveat qualifies these implications. The interventions synthesized in this review primarily operated at the level of the individual nurse by strengthening coping, psychological flexibility, stress recovery, or self-management. They did not directly modify the organizational conditions under which burnout develops, including workload, staffing, job control, reward, and role clarity. This distinction is consistent with the JD-R model and with long-standing burnout theory, both of which locate burnout in the interaction between individual resources and chronic workplace demands (31–34). The moderate effects observed here should therefore be understood as evidence that DHIs can complement organizational reform, not replace it. If digital well-being platforms are used instead of adequate staffing, workload redesign, or schedule control, they risk shifting responsibility for a systemic problem onto individual nurses.

### 4.7 Exploring the sources of heterogeneity

The substantial heterogeneity observed in the primary analysis requires explicit interpretation because it affects the precision and generalizability of the pooled estimate. This dispersion is unlikely to be explained by statistical noise alone. It appears to reflect clinical and methodological diversity within a deliberately broad evidence base, which the pre-specified subgroup analyses resolved only in part.

Several clinical sources are plausible contributors. First, intervention content and therapeutic framework varied widely. Structured CBT and ACT programs differ substantially from unguided commercial mindfulness applications, even though both are delivered digitally. Modality accounted for a significant share of between-study variation, and within-subgroup heterogeneity was lower for some more homogeneous categories. Second, intervention intensity and duration differed markedly, ranging from a single VR relaxation session to 12-week multi-module programs. Although the formal subgroup test by duration was not statistically significant, the observed ordering of effects was consistent with the possibility that intervention dose contributes to between-study variation. Third, baseline burnout severity and work context varied across studies. Trials conducted during acute COVID-19 surges may have enrolled nurses under systemic strain that affected both baseline burnout and the achievable degree of improvement (3, 40–42).

Methodological diversity also contributed to heterogeneity. Quasi-experimental studies were pooled alongside randomized and cluster-randomized trials, although sensitivity analysis restricted to randomized evidence preserved the direction and approximate magnitude of the pooled effect. Comparator conditions also varied, ranging from waitlist or no-intervention controls to active and face-to-face comparators. In addition, burnout was measured using different instruments, including the MBI, CBI, BAT, and ProQOL, whose subscale structures and scoring systems are not fully interchangeable (1, 17–19). Outcome timing also differed, ranging from immediate post-intervention assessment to follow-up several months later.

These observations indicate that the pooled SMD should be read as an average across a heterogeneous evidence base rather than as the expected effect of any specific intervention. The modality-specific, duration-specific, and guidance-specific estimates are therefore more clinically informative than the overall estimate alone. Residual unexplained heterogeneity is reflected in the GRADE assessment, particularly in the downgrading of certainty for overall burnout and emotional exhaustion (24).

### 4.8 Limitations

Several limitations should be considered. First, substantial heterogeneity was observed in the primary analysis, reflecting variation in DHI modality, therapeutic framework, intervention duration, comparator type, burnout measure, study design, and participant population. Although subgroup and sensitivity analyses explored several sources of heterogeneity, residual unexplained variability limits the precision of modality-specific estimates.

Second, all included studies relied on self-reported burnout measures. Because participant blinding is difficult in behavioral DHIs, outcome measurement may have been influenced by expectation effects, social desirability, or awareness of intervention assignment. No study incorporated objective stress biomarkers, such as cortisol, heart rate variability, or sleep-derived indicators, as co-primary outcomes.

Third, intervention engagement varied substantially, and many studies did not report adherence or completion in sufficient detail. This limits understanding of how dose, engagement, and intervention effects are connected. Fourth, the evidence base was geographically concentrated in high-income Western and East Asian countries, with only one study from a low- or middle-income country. Findings should therefore not be assumed to generalize to settings with different staffing pressures, digital infrastructure, occupational risks, and resource constraints. Digital mental health research in low- and middle-income countries also suggests that implementation may require locally adapted, low-bandwidth, and culturally appropriate platforms (43).

Fifth, the review population was clinically heterogeneous. Registered nurses, licensed practical nurses, nursing assistants, and other nursing staff differ in scope of practice, job control, responsibility, and exposure to patient-care demands. Reporting was too inconsistent to permit subgroup analysis by professional role, and future trials should stratify or report outcomes by nursing category.

Sixth, only English-language studies were included. Non-English records were excluded during screening but were not tabulated as a separate exclusion category, creating a reporting limitation and possible language bias. Seventh, the search strategy was not formally reviewed by a librarian or information specialist using PRESS criteria, although it was refined through pilot testing and cross-checking against known eligible studies.

Eighth, follow-up durations were generally short, and evidence on the durability of DHI effects after intervention cessation remains limited. Ninth, the AI-tailored intervention evidence came from only three studies from a single research group, limiting generalizability. Finally, although Egger's test was not statistically significant, funnel plot asymmetry and trim-and-fill results suggest that small-study effects may have modestly inflated the pooled estimate. The adjusted estimate, however, remained statistically significant.

### 4.9 Future research directions

Future research should move beyond whether DHIs reduce nurse burnout in general and examine which digital formats work best, for whom, and under what conditions. First, head-to-head comparative effectiveness trials are needed to directly compare web-based CBT/ACT programs, AI-tailored applications, VR interventions, and app-based mindfulness programs within the same nursing populations. Second, larger multicenter RCTs with active comparators, pre-registered protocols, and follow-up periods of at least 12 months are needed to establish the durability and comparative value of DHI effects.

Third, future trials should combine validated burnout instruments with objective or passive indicators, such as heart rate variability, sleep quality, or other wearable-derived measures, to reduce reliance on self-report alone. Fourth, implementation-science studies using hybrid effectiveness–implementation designs are needed to identify barriers and facilitators to sustained DHI adoption in real-world healthcare organizations.

Fifth, future interventions should examine whether digital tools can address organizational-level burnout determinants, such as scheduling, staffing strain, leadership support, and workload management, rather than focusing only on individual coping. Finally, equity-focused research is needed to evaluate DHI effectiveness across nursing roles, clinical specialties, career stages, demographic groups, and geographic settings, particularly in low- and middle-income countries (43).

## 5 Conclusions

This systematic review and meta-analysis of 37 controlled studies found that DHIs were associated with a statistically significant moderate reduction in occupational burnout among nurses and nursing staff. Effects varied substantially by modality, delivery format, and burnout dimension. Web-based CBT/ACT programs showed the strongest current evidence, guided delivery outperformed fully self-guided formats, and AI-tailored interventions showed promising early effects that require independent replication. Emotional exhaustion was the most responsive dimension, while personal accomplishment remained the most resistant to change.

These findings support the integration of evidence-based DHIs into comprehensive workforce well-being strategies. The strongest case is for structured, guided, and theory-based programs that combine digital scalability with human support. However, digital tools should complement, not replace, organizational reforms addressing workload, staffing, autonomy, recognition, and role clarity. Technology can extend access to support, but sustainable burnout prevention in nursing still requires attention to the organizational conditions under which care work is performed.

## Data Availability

The original contributions presented in the study are included in the article/Supplementary material, further inquiries can be directed to the corresponding author.
