# Supplementary material for: Digital health interventions for reducing occupational burnout in nurses: a systematic review and meta-analysis
Source: Front Public Health. 2026 Aug 24;14:1879258. doi: 10.3389/fpubh.2026.1879258 (PMC13547474; doi:10.3389/fpubh.2026.1879258)
Supplement: Supplementary file 1 [file Table_1.docx]

# Appendix S1 — Complete Search Strategy for Each Database

**Manuscript**: Digital Health Interventions for Reducing Occupational Burnout in Nurses: A Systematic Review and Meta-Analysis

**Search dates**: Database inception to March 15, 2025 (lower bound restricted to January 2015 to capture contemporary smartphone-based digital health interventions).

**Languages**: No language restrictions at the search stage. Non-English records were excluded at the screening stage.

## 1. Conceptual Search Blocks

The search strategy combined three concept blocks using Boolean operators (Block 1 AND Block 2 AND Block 3).

### Block 1 — Digital Health Interventions

"digital health" OR "mHealth" OR "eHealth" OR "mobile health"
OR "mobile application*" OR "smartphone app*"
OR "web-based intervention" OR "web-based program"
OR "online intervention" OR "internet-based"
OR "telehealth" OR "telemedicine"
OR "virtual reality" OR "augmented reality"
OR "wearable device*" OR "biofeedback"
OR "artificial intelligence" OR "chatbot" OR "conversational agent"
OR "digital therapeutics"

### Block 2 — Burnout

"burnout" OR "burn-out"
OR "occupational burnout" OR "professional burnout"
OR "emotional exhaustion" OR "depersonalization"
OR "compassion fatigue"

### Block 3 — Nurses

"nurse*" OR "nursing"
OR "nursing staff" OR "nursing personnel"
OR "registered nurse*"
OR "licensed practical nurse*"

## 2. Database-Specific Translations

### 2.1 PubMed / MEDLINE

```
((("digital health"[MeSH Terms] OR "telemedicine"[MeSH Terms]
  OR "mobile applications"[MeSH Terms] OR "wearable electronic devices"[MeSH Terms]
  OR "virtual reality"[MeSH Terms] OR "artificial intelligence"[MeSH Terms])
 OR ("digital health"[Title/Abstract] OR "mHealth"[Title/Abstract]
  OR "eHealth"[Title/Abstract] OR "mobile health"[Title/Abstract]
  OR "telehealth"[Title/Abstract] OR "web-based intervention*"[Title/Abstract]
  OR "smartphone app*"[Title/Abstract] OR "wearable*"[Title/Abstract]
  OR "virtual reality"[Title/Abstract] OR "artificial intelligence"[Title/Abstract]
  OR "chatbot*"[Title/Abstract]))
AND
("burnout, professional"[MeSH Terms]
 OR "burnout"[Title/Abstract] OR "burn-out"[Title/Abstract]
 OR "emotional exhaustion"[Title/Abstract] OR "depersonalization"[Title/Abstract]
 OR "compassion fatigue"[Title/Abstract])
AND
("nurses"[MeSH Terms] OR "nursing staff"[MeSH Terms]
 OR "nurse*"[Title/Abstract] OR "nursing"[Title/Abstract]))
AND ("2015/01/01"[Date - Publication] : "2025/03/15"[Date - Publication])
```

### 2.2 CINAHL Complete (via EBSCOhost)

```
((MH "Digital Health" OR MH "Telehealth" OR MH "Mobile Applications"
  OR MH "Wearable Devices" OR MH "Virtual Reality" OR MH "Artificial Intelligence")
 OR (TI ("digital health" OR mHealth OR eHealth OR "mobile health"
   OR telehealth OR "web-based intervention*" OR "smartphone app*"
   OR wearable* OR "virtual reality" OR "artificial intelligence" OR chatbot*)
  OR AB ("digital health" OR mHealth OR eHealth OR "mobile health"
   OR telehealth OR "web-based intervention*" OR "smartphone app*"
   OR wearable* OR "virtual reality" OR "artificial intelligence" OR chatbot*)))
AND
((MH "Burnout, Professional")
 OR (TI (burnout OR "burn-out" OR "emotional exhaustion" OR depersonalization
   OR "compassion fatigue")
  OR AB (burnout OR "burn-out" OR "emotional exhaustion" OR depersonalization
   OR "compassion fatigue")))
AND
((MH "Nurses+" OR MH "Nursing Staff+")
 OR (TI (nurse* OR nursing OR "registered nurse*")
  OR AB (nurse* OR nursing OR "registered nurse*")))
Limit: 2015–2025
```

### 2.3 Embase (via Ovid)

```
1.  exp digital health/ OR exp telemedicine/ OR exp mobile application/
    OR exp virtual reality/ OR exp artificial intelligence/
    OR exp wearable device/
2.  ("digital health" OR mHealth OR eHealth OR "mobile health"
    OR telehealth OR "web-based intervention*" OR "smartphone app*"
    OR wearable* OR "virtual reality" OR "artificial intelligence"
    OR chatbot*).ti,ab.
3.  1 OR 2
4.  exp burnout/ OR exp professional burnout/
5.  (burnout OR "burn-out" OR "emotional exhaustion" OR depersonalization
    OR "compassion fatigue").ti,ab.
6.  4 OR 5
7.  exp nurse/ OR exp nursing staff/
8.  (nurse* OR nursing OR "registered nurse*").ti,ab.
9.  7 OR 8
10. 3 AND 6 AND 9
11. limit 10 to (yr="2015–2025")
```

### 2.4 Web of Science Core Collection

```
TS=(("digital health" OR mHealth OR eHealth OR "mobile health"
   OR telehealth OR "web-based intervention*" OR "smartphone app*"
   OR wearable* OR "virtual reality" OR "artificial intelligence" OR chatbot*))
AND
TS=((burnout OR "burn-out" OR "emotional exhaustion" OR depersonalization
   OR "compassion fatigue"))
AND
TS=((nurse* OR nursing OR "registered nurse*"))
Timespan: 2015–2025
Document types: Article, Review, Proceedings Paper
```

### 2.5 PsycINFO (via APA PsycNET)

```
(DE "Digital Health" OR DE "Telemedicine" OR DE "Mobile Devices"
 OR DE "Virtual Reality" OR DE "Artificial Intelligence")
OR
TI,AB ("digital health" OR mHealth OR eHealth OR "mobile health"
  OR telehealth OR "web-based intervention*" OR "smartphone app*"
  OR wearable* OR "virtual reality" OR "artificial intelligence" OR chatbot*)
AND
(DE "Occupational Stress" OR DE "Burnout" OR DE "Emotional Exhaustion")
OR
TI,AB (burnout OR "burn-out" OR "emotional exhaustion" OR depersonalization
  OR "compassion fatigue")
AND
(DE "Nurses" OR DE "Nursing Staff")
OR
TI,AB (nurse* OR nursing OR "registered nurse*")
Limit: 2015–2025
```

### 2.6 Scopus

```
TITLE-ABS-KEY (
  ( "digital health" OR mHealth OR eHealth OR "mobile health"
    OR telehealth OR "web-based intervention*" OR "smartphone app*"
    OR wearable* OR "virtual reality" OR "artificial intelligence" OR chatbot* )
  AND
  ( burnout OR "burn-out" OR "emotional exhaustion" OR depersonalization
    OR "compassion fatigue" )
  AND
  ( nurse* OR nursing OR "registered nurse*" )
)
AND PUBYEAR > 2014 AND PUBYEAR < 2026
AND ( LIMIT-TO ( DOCTYPE , "ar" ) OR LIMIT-TO ( DOCTYPE , "re" ) )
```

## 3. Supplementary Search Methods

In addition to the database searches above, the following supplementary methods were used:

1. **Backward reference searching** — All reference lists of included studies and topically adjacent reviews were screened for potentially eligible studies missed in the database search.
2. **Forward citation tracking** — All included studies were forward-searched in Google Scholar and Web of Science for subsequent citing articles, with a citation cap of 200 records per source study.
3. **Hand-searching of key journals** (January 2022 – March 2025):
   - Journal of Medical Internet Research
   - JMIR mHealth and uHealth
   - JMIR Mental Health
   - Digital Health
4. **Grey literature** — ClinicalTrials.gov and the ISRCTN registry were screened for completed but unpublished trials matching the eligibility criteria.

## 4. Search Yield Summary

| Source | Records identified |
| --- | --- |
| PubMed / MEDLINE | (TBD) |
| CINAHL Complete | (TBD) |
| Embase | (TBD) |
| Web of Science Core Collection | (TBD) |
| PsycINFO | (TBD) |
| Scopus | (TBD) |
| Backward / forward citation searching | (TBD) |
| Hand-searched journals | (TBD) |
| Grey literature | (TBD) |
| **Total records identified** | **2,847** |
| Duplicates removed | 682 |
| Records screened (title/abstract) | 2,165 |
| Records excluded at screening | 2,043 |
| Full texts retrieved | 122 |
| Full texts excluded (Appendix S2) | 85 |
| **Studies included in qualitative synthesis** | **37** |
| Studies included in meta-analysis | 28 |

**Note for authors**: replace the (TBD) rows with the per-database record counts produced by your final search export before submission.
