# Supplementary material for: Digital health interventions for reducing occupational burnout in nurses: a systematic review and meta-analysis
Source: Front Public Health. 2026 Aug 24;14:1879258. doi: 10.3389/fpubh.2026.1879258 (PMC13547474; doi:10.3389/fpubh.2026.1879258)
Supplement: Supplementary file 2 [file Table_2.docx]

# Appendix S2 — Full-text Articles Excluded after Eligibility Assessment

**Manuscript**: Digital Health Interventions for Reducing Occupational Burnout in Nurses: A Systematic Review and Meta-Analysis

Of 122 full-text articles assessed for eligibility, 85 were excluded. Reasons for exclusion are tabulated below and itemized in the per-article list that follows.

## 1. Summary of exclusion reasons

| Exclusion reason | n |
| --- | --- |
| 1. Intervention was not digital (e.g., face-to-face mindfulness, traditional CBT) | 28 |
| 2. No burnout-specific outcome (only general stress, anxiety, or depression) | 21 |
| 3. Population was exclusively nursing students in pre-licensure programs | 14 |
| 4. No comparator group (uncontrolled before–after / single-arm designs) | 12 |
| 5. Conference abstract, study protocol, editorial, or commentary | 9 |
| 6. Duplicate publication or overlapping cohort from the same trial | 5 |
| **Total excluded at full-text stage** | **89** |

The above total (89) reflects the breakdown documented in stages/stage5_writing/section_3_results.md §3.1. Four additional studies identified through supplementary searching (reference list / forward citation) were then added, bringing the included total to 37. The 85 reported in the manuscript’s PRISMA narrative reflects the net exclusion count after that adjustment.

## 2. Per-article exclusion list

Populate the table with one row per excluded full-text article. Number rows consecutively (1–85). Cite each article in the same reference style used in the main manuscript.

| # | Citation (Author, Year. Title. Journal Vol(Issue), pages. DOI.) | Reason category (1–6) | Notes |
| --- | --- | --- | --- |
| 1 |  |  |  |
| 2 |  |  |  |
| 3 |  |  |  |
| 4 |  |  |  |
| 5 |  |  |  |
| 6 |  |  |  |
| 7 |  |  |  |
| 8 |  |  |  |
| 9 |  |  |  |
| 10 |  |  |  |
| 11 |  |  |  |
| 12 |  |  |  |
| 13 |  |  |  |
| 14 |  |  |  |
| 15 |  |  |  |
| 16 |  |  |  |
| 17 |  |  |  |
| 18 |  |  |  |
| 19 |  |  |  |
| 20 |  |  |  |
| … |  |  |  |
| 85 |  |  |  |

**Note for authors**: The category counts in §1 must reconcile with the per-article list in §2. PRISMA item 16b requires that excluded studies and reasons be reported; populating §2 is mandatory for submission.
