# Supplementary material for: Digital health interventions for reducing occupational burnout in nurses: a systematic review and meta-analysis
Source: Front Public Health. 2026 Aug 24;14:1879258. doi: 10.3389/fpubh.2026.1879258 (PMC13547474; doi:10.3389/fpubh.2026.1879258)
Supplement: Supplementary file 3 [file Table_3.docx]

# Appendix S3 — GRADE Evidence Profiles

**Manuscript**: Digital Health Interventions for Reducing Occupational Burnout in Nurses: A Systematic Review and Meta-Analysis

GRADE certainty was assessed independently by two reviewers and finalized by consensus across five domains: risk of bias, inconsistency, indirectness, imprecision, and publication bias. The body of evidence for each outcome derives from randomized controlled trials and controlled quasi-experimental studies; the starting certainty for each outcome is therefore *High*, then downgraded for each serious or very serious concern.

## 1. Summary of findings

| Outcome | Studies (k) | Participants (N) | SMD (95% CI) | Certainty | Interpretation |
| --- | --- | --- | --- | --- | --- |
| Overall burnout | 28 | 6,820 | −0.47 (−0.65, −0.29) | ⊕⊕⊕◯ Moderate | DHIs probably produce a moderate reduction in burnout |
| Emotional exhaustion (EE) | 22 | 5,640 | −0.53 (−0.74, −0.32) | ⊕⊕⊕◯ Moderate | DHIs probably produce a moderate reduction in EE |
| Depersonalization (DP) | 22 | 5,640 | −0.38 (−0.56, −0.20) | ⊕⊕◯◯ Low | DHIs may produce a small-to-moderate reduction in DP |
| Personal accomplishment (PA) | 22 | 5,640 | +0.24 (0.05, 0.43) | ⊕⊕◯◯ Low | DHIs may produce a small improvement in PA |

## 2. Detailed evidence profile

### 2.1 Overall burnout

| Domain | Judgment | Rationale |
| --- | --- | --- |
| Study limitations (risk of bias) | Serious (−1) | Blinding of participants is infeasible in behavioral DHIs; outcome assessment relies on self-reported burnout instruments. 13 of 24 RCTs were rated as having “some concerns” on RoB 2 Domain 2 (deviations from intended interventions) and Domain 4 (outcome measurement). |
| Inconsistency | Not serious | Heterogeneity is substantial (I² = 72%) but plausibly explained by intervention modality on subgroup analysis (web-based CBT/ACT vs app-based mindfulness; p = .04). |
| Indirectness | Not serious | All studies enrolled clinically practising nurses; outcomes were validated burnout instruments aligned with the review question. |
| Imprecision | Not serious | 95% CI for the pooled SMD does not cross the null and excludes a trivial effect (upper bound −0.29). Optimal information size threshold met (N = 6,820 > 4,000). |
| Publication bias | Not detected | Funnel plot showed slight asymmetry but Egger’s regression test was non-significant (p = .12). |
| **Overall certainty** | **⊕⊕⊕◯ Moderate** | Single downgrade for risk of bias. |

### 2.2 Emotional exhaustion (EE)

| Domain | Judgment | Rationale |
| --- | --- | --- |
| Study limitations | Serious (−1) | Same blinding and measurement concerns as for overall burnout. EE is the most commonly reported MBI subscale and shares the dominant detection-bias risk. |
| Inconsistency | Not serious | I² = 69%; heterogeneity attenuates when restricted to RCTs of guided web-based programs. |
| Indirectness | Not serious | EE measured with MBI in all contributing studies. |
| Imprecision | Not serious | 95% CI −0.74 to −0.32 excludes the null and a trivial effect. |
| Publication bias | Not detected | Symmetric funnel for EE-specific subgroup. |
| **Overall certainty** | **⊕⊕⊕◯ Moderate** | Single downgrade for risk of bias. |

### 2.3 Depersonalization (DP)

| Domain | Judgment | Rationale |
| --- | --- | --- |
| Study limitations | Serious (−1) | Same blinding and measurement concerns; DP responses are particularly susceptible to social desirability bias when reported on unblinded self-report instruments. |
| Inconsistency | Serious (−1) | I² = 54% with effect sizes ranging from null to moderate across modalities; modality-stratified subgroups remained heterogeneous. |
| Indirectness | Not serious | DP measured with MBI in all contributing studies. |
| Imprecision | Not serious | 95% CI −0.56 to −0.20 excludes the null. |
| Publication bias | Not detected | Insufficient studies for formal small-study test; visual inspection unremarkable. |
| **Overall certainty** | **⊕⊕◯◯ Low** | Two downgrades (risk of bias + inconsistency). |

### 2.4 Personal accomplishment (PA)

| Domain | Judgment | Rationale |
| --- | --- | --- |
| Study limitations | Serious (−1) | Same self-report concerns. PA is the MBI subscale most often reported only as a secondary outcome with less methodological focus. |
| Inconsistency | Not serious | I² = 61% but direction of effect is consistent across studies (positive). |
| Indirectness | Not serious | PA measured with MBI in all contributing studies. |
| Imprecision | Serious (−1) | 95% CI 0.05 to 0.43 narrowly excludes the null; the lower bound borders the threshold for a trivial effect. |
| Publication bias | Not detected | Insufficient studies for formal test; visual inspection unremarkable. |
| **Overall certainty** | **⊕⊕◯◯ Low** | Two downgrades (risk of bias + imprecision). |

## 3. Notes on certainty interpretation

- **⊕⊕⊕⊕ High** — We are very confident that the true effect lies close to the estimate.
- **⊕⊕⊕◯ Moderate** — We are moderately confident; the true effect is likely to be close to the estimate, but may be substantially different.
- **⊕⊕◯◯ Low** — Our confidence is limited; the true effect may be substantially different.
- **⊕◯◯◯ Very Low** — We have very little confidence in the effect estimate.

**Note for authors**: These profiles are derived from the summary in Section 3.6 (Table 2) of the manuscript plus the methodological detail in Section 2.5–2.6. If your final analysis updates k, N, or effect sizes, refresh the §1 summary table and re-justify each judgment in §2 accordingly. Recommended downgrade-rationale phrasing follows the GRADE Handbook (Schünemann et al., 2013).
