# Supplementary material for: Digital health interventions for reducing occupational burnout in nurses: a systematic review and meta-analysis
Source: Front Public Health. 2026 Aug 24;14:1879258. doi: 10.3389/fpubh.2026.1879258 (PMC13547474; doi:10.3389/fpubh.2026.1879258)
Supplement: Supplementary file 4 [file Table_4.docx]

# File S1 — PRISMA 2020 Checklist

Reference: Page MJ, McKenzie JE, Bossuyt PM, et al. The PRISMA 2020 statement: an updated guideline for reporting systematic reviews. BMJ 2021;372:n71. https://doi.org/10.1136/bmj.n71

**Manuscript**: Digital Health Interventions for Reducing Occupational Burnout in Nurses: A Systematic Review and Meta-Analysis

For each item, indicate the page number(s) of the manuscript where the item is reported, or N/A if the item is not applicable to this review.

## Title

| # | Item | Reported on page(s) |
| --- | --- | --- |
| 1 | Identify the report as a systematic review. | 1 |

## Abstract

| # | Item | Reported on page(s) |
| --- | --- | --- |
| 2 | See the PRISMA 2020 for Abstracts checklist (Table 2 of the PRISMA 2020 statement). | 2 |

## Introduction

| # | Item | Reported on page(s) |
| --- | --- | --- |
| 3 | Describe the rationale for the review in the context of existing knowledge. | __ |
| 4 | Provide an explicit statement of the objective(s) or question(s) the review addresses. | __ |

## Methods

| # | Item | Reported on page(s) |
| --- | --- | --- |
| 5 | Specify the inclusion and exclusion criteria for the review and how studies were grouped for the syntheses. | __ |
| 6 | Specify all databases, registers, websites, organisations, reference lists and other sources searched or consulted. | __ |
| 7 | Present the full search strategies for all databases, registers and websites, including any filters and limits used. | __ |
| 8 | Specify the methods used to decide whether a study met the inclusion criteria of the review. | __ |
| 9 | Specify the methods used to collect data from reports. | __ |
| 10a | List and define all outcomes for which data were sought. | __ |
| 10b | List and define all other variables for which data were sought. | __ |
| 11 | Specify the methods used to assess risk of bias in the included studies. | __ |
| 12 | Specify the effect measure(s) used in the synthesis or presentation of results. | __ |
| 13a | Describe the processes used to decide which studies were eligible for each synthesis. | __ |
| 13b | Describe any methods required to prepare the data for presentation or synthesis. | __ |
| 13c | Describe any methods used to tabulate or visually display results of individual studies and syntheses. | __ |
| 13d | Describe any methods used to synthesise results and provide a rationale for the choice(s). | __ |
| 13e | Describe any methods used to explore possible causes of heterogeneity among study results. | __ |
| 13f | Describe any sensitivity analyses conducted to assess robustness of the synthesised results. | __ |
| 14 | Describe any methods used to assess risk of bias due to missing results in a synthesis. | __ |
| 15 | Describe any methods used to assess certainty (or confidence) in the body of evidence for an outcome. | __ |

## Results

| # | Item | Reported on page(s) |
| --- | --- | --- |
| 16a | Describe the results of the search and selection process. | __ |
| 16b | Cite studies that might appear to meet the inclusion criteria but which were excluded, and explain why. | __ |
| 17 | Cite each included study and present its characteristics. | __ |
| 18 | Present assessments of risk of bias for each included study. | __ |
| 19 | For all outcomes, present, for each study: (a) summary statistics for each group and (b) an effect estimate and its precision. | __ |
| 20a | For each synthesis, briefly summarise the characteristics and risk of bias among contributing studies. | __ |
| 20b | Present results of all statistical syntheses conducted. | __ |
| 20c | Present results of all investigations of possible causes of heterogeneity among study results. | __ |
| 20d | Present results of all sensitivity analyses conducted to assess robustness of the synthesised results. | __ |
| 21 | Present assessments of risk of bias due to missing results for each synthesis. | __ |
| 22 | Present assessments of certainty (or confidence) in the body of evidence for each outcome. | __ |

## Discussion

| # | Item | Reported on page(s) |
| --- | --- | --- |
| 23a | Provide a general interpretation of the results in the context of other evidence. | __ |
| 23b | Discuss any limitations of the evidence included in the review. | __ |
| 23c | Discuss any limitations of the review processes used. | __ |
| 23d | Discuss implications of the results for practice, policy and future research. | __ |

## Other information

| # | Item | Reported on page(s) |
| --- | --- | --- |
| 24a | Provide registration information for the review, including register name and registration number, or state that the review was not registered. | __ |
| 24b | Indicate where the review protocol can be accessed, or state that a protocol was not prepared. | __ |
| 24c | Describe and explain any amendments to information provided at registration or in the protocol. | __ |
| 25 | Describe sources of financial or non-financial support for the review, and the role of the funders or sponsors in the review. | __ |
| 26 | Declare any competing interests of review authors. | __ |
| 27 | Report which of the following are publicly available and where they can be found: template data collection forms; data extracted from included studies; data used for all analyses; analytic code; any other materials used in the review. | __ |

**Note for authors**: fill in the page numbers from the typeset manuscript (or the manuscript PDF you upload to Frontiers) before submission. Items marked __ must all be completed; items reported as N/A should include a brief reason.
